# A 30-yr high-resolution weather research and forecasting model downscaling data over California and Nevada

**DOI:** 10.1016/j.dib.2025.111562

**Published:** 2025-04-21

**Authors:** Charles Jones, Donald D. Lucas, Allison Bagley, Callum Thompson

**Affiliations:** a Department of Geography, University of California Santa Barbara, CA 93106-3060, USA; b Atmospheric, Earth, and Energy Division, Lawrence Livermore National Laboratory, Livermore, CA 94550, USA; c Earth Research Institute, University of California Santa Barbara, CA 93106-3060, USA

**Keywords:** Weather research and forecasting model, Dynamical downscaling, Fire weather, Gridded meteorological dataset, Meteorology, Climate, Regional climate, Wildfires

## Abstract

This dataset presents a 30-year high resolution meteorological dataset obtained using the WRF model (Advanced version Research WRF version 4.4). We used WRF and European Centre for Medium-Range Weather Forecasts Reanalysis v5 as initial and boundary conditions to generate gridded meteorological variables. A large number of surface weather stations was used for model validation. A multi-physics analysis was first developed to identify a good physics suite extended from 6 November 00 UTC to 10 November 23 UTC, 2018, which included the Camp Fire in northern California. Based on the best physics suite, the downscaling dataset extends from 1 December to 28 February, 1990–2021 and the horizontal domain has 1.5 km grid spacing covering the entire states of California and Nevada in the United States. Comparisons between hourly surface observations and WRF simulations of air temperature, relative humidity and wind speeds show mean absolute errors on the order of (1.6-2.0 C), (10 %) and 1.2–1.5 m s^-1^, respectively.

Specifications Table

**Table:** 

Subject	Earth & Environmental Sciences
Specific subject area	30-year, 1.5 km grid spacing, hourly, dynamical downscaling with the Weather Research and Forecasting (WRF) model over California and Nevada, United States
Type of data	Gridded meteorological data in netcdf format; README text files*.*
Data collection	The data were produced with the Weather Research and Forecasting (WRF) model with initial and boundary conditions from ERA5 reanalysis. WRF configuration was based on results from multi-physics simulations during a 5-day period. Downscaling was produced from 1 December to 28 February, 1990–2021, 1.5 km horizontal grid spacing, hourly, covering the entire states of California and Nevada in the United States. WRF downscaling data were validated with observations from over 2400 weather stations.
Data source location	University of California Santa Barbara, California, United States*.*
Data accessibility	Repository name: World Data Climate Center Data identification number: 10.26050/WDCC/WRF_CALNEV_v1 Direct URL to data: lead author’s public ftp site: https://nextcloud.grit.ucsb.edu/index.php/s/mX4scc88xPjJsPe
Related research article	None*.*

## 1 Value of the Data

• The WRF dynamical downscaling dataset extends over a 30-year period. It has high spatiotemporal resolutions (1.5 km grid spacing, hourly) and covers the states of California and Nevada in the United States.
• The dataset is invaluable for studying atmospheric variability over the complex terrains of California and Nevada, where rugged topography and atmospheric boundary layer interactions produce diverse weather patterns and microclimates [1].
• This dataset enables investigations of downslope windstorms [2], mountain wave activity, renewable energy planning, wind resource assessment for wind farm siting, and microclimates that affect renewable energy potential.
• Extending over the Pacific, the WRF downscaling domain provides insights into ocean-atmospheric processes off the coast of California. The high spatiotemporal resolution data can serve as initial and boundary conditions for regional ocean models such as the Regional Ocean Modeling System (ROMS).
• The dataset is suitable for risk analyses, including probabilities of high winds and fire-weather events [[3], [4], [5]]. WRF outputs can be integrated with uncoupled fire spread models [6,5].
• The high-resolution of the WRF dataset can be used to train machine learning applications to downscale coarse global datasets.

## 2 Background

The complex topography of California plays a major role in generating weather occurrences on micro, mesoscale and synoptic scales [7,8]. Oftentimes, some weather types can lead to hazardous conditions with unforeseen consequences. Thus, understanding physical processes, monitoring and predicting these weather types is critical to develop mitigation and adaptation strategies. Meteorological datasets with sufficient spatiotemporal resolutions are critical for resolving the scales of the atmospheric phenomena under investigation.

We present a 30-year high resolution meteorological dataset obtained using the WRF model (Advanced version Research WRF version 4.4) [9]. WRF is a community model capable of simulating the evolution of the atmosphere on tens of meters to global scales (https://www.mmm.ucar.edu/models/wrf). It offers a wide range of numerical parameterizations to represent physical processes associated with atmospheric radiation transfer, cumulus convection, cloud microphysics, the planetary boundary layer, and land-atmosphere interactions. We used WRF and ERA5 reanalysis as initial and boundary conditions to generate gridded meteorological variables with 1.5 km horizontal grid spacing 1 December to 28 February, 1990–2021. The downscaling did not employ observational nudging via a four-dimensional data assimilation (FDDA) system. Rather, a common procedure is to employ nudging towards the reanalysis to ensure that the high-resolution model state remains close to the reanalysis [10].

## 3 Data Description

The dataset, named WRF-CALNEV, (https://doi.org/10.26050/WDCC/WRF_CALNEV_v1) [11] is available at the World Data Climate Center. Table 1 describes the data records. Note that the total size for air temperature at 2 m (TA), wind speed at 10 m (WS) and relative humidity at 2 m (RH) is about 2 TB and, therefore, only this limited set of variables is available in the current release. Users interested in accessing additional WRF variables or the entire raw downscaling dataset need to contact the authors for additional instructions. A README file is available in the repository explaining directory structure, filenames, file sizes and other information.

**Table 1:** WRF-CALNEV data records.

Description	Filename	Variables	Units Format	Total Size
Static file generated by the WPS-geogrid program for the 7.5 km outer domain	geo_em.d01.nc	Standard output variables from WPS program	netcdf	98 MB
Static file generated by the WPS-geogrid program for the 1.5 km inner domain	geo_em.d02.nc	Standard output variables from WPS program	netcdf	69 MB
Hourly TA at 2 m for the 1.5 km inner domain. Files are in four subfolders: djf, mam, jja, son. Each file contains variable and coordinates	wrfout_d02_V1_TA_YYY1-MO1-DAY1_YYY2-MO2-DY2.nc where YYY1-MO1-DY1 and YYY2-MO2-DY2 are beginnig and ending dates of the season	airtemp, XLAT, XLONG	Celsius netcdf	286 GB 282 GB 282 GB 279 GB
Hourly WS at 10 m for the 1.5 km inner domain. Files are in four subfolders: djf, mam, jja, son. Each file contains variable and coordinates	wrfout_d02_V1_WS_YYY1-MO1-DAY1_YYY2-MO2-DY2.nc where YYY1-MO1-DY1 and YYY2-MO2-DY2 are beginnig and ending dates of the season	wspeed, XLAT, XLONG	m /s netcdf	286 GB 282 GB 282 GB 279 GB
Hourly RH at 2 m for the 1.5 km inner domain.Files are in four subfolders: djf, mam, jja, son. Each file contains variable and coordinates	wrfout_d02_V1_RH_YYY1-MO1-DAY1_YYY2-MO2-DY2.nc where YYY1-MO1-DY1 and YYY2-MO2-DY2 are beginnig and ending dates of the season	re;hum, XLAT, XLONG	% netcdf	286 GB 282 GB 282 GB 279 GB

The static files geo_em_d01.nc and geo_em_d02.nc are generated by the WRF Pre-processing (WPS) program and contain standard terrain information for the 7.5 km and 1.5 km grids, respectively. Additional information about the WPS program is available at https://www2.mmm.ucar.edu/wrf/users/.

**Fig. 1:**
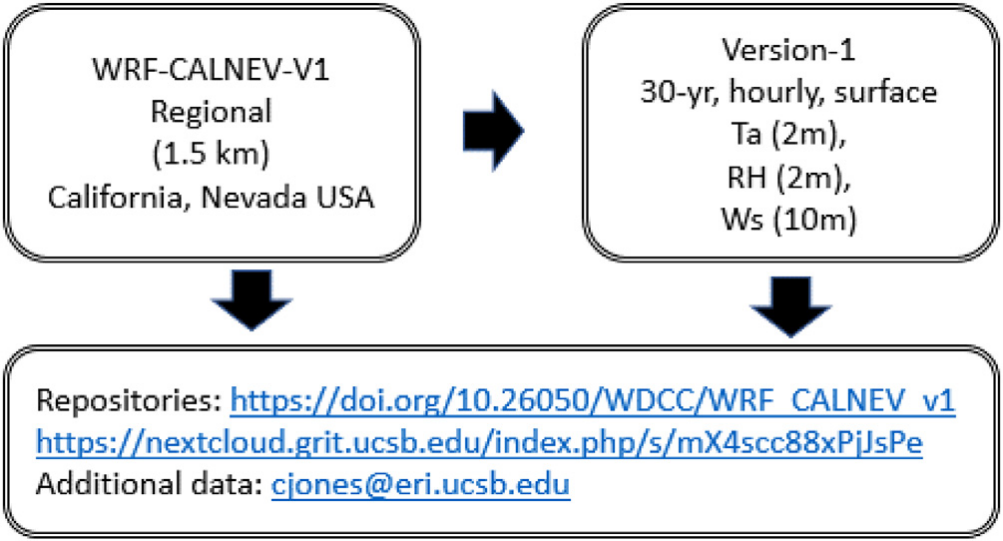
Flowchart describing the WRF-CALNEV dataset.

Hourly TA, WS and RH for the period 1 December – 28 February, 1990–2021 have been extracted from the original WRF output files using the python programming language. Files for each variable are available in seasons separately. File format is in the UNIDATA Network Common Data Form (NetCDF) (https://www.unidata.ucar.edu/software/netcdf/) and can be accessed with a variety of programming languages. For example, python xarrays (https://docs.xarray.dev/en/stable/) is an efficient way of accessing the data, metadata and visualizing the variables. Fig. 1 shows a flowchart of data and repository.

**Fig. 2:**
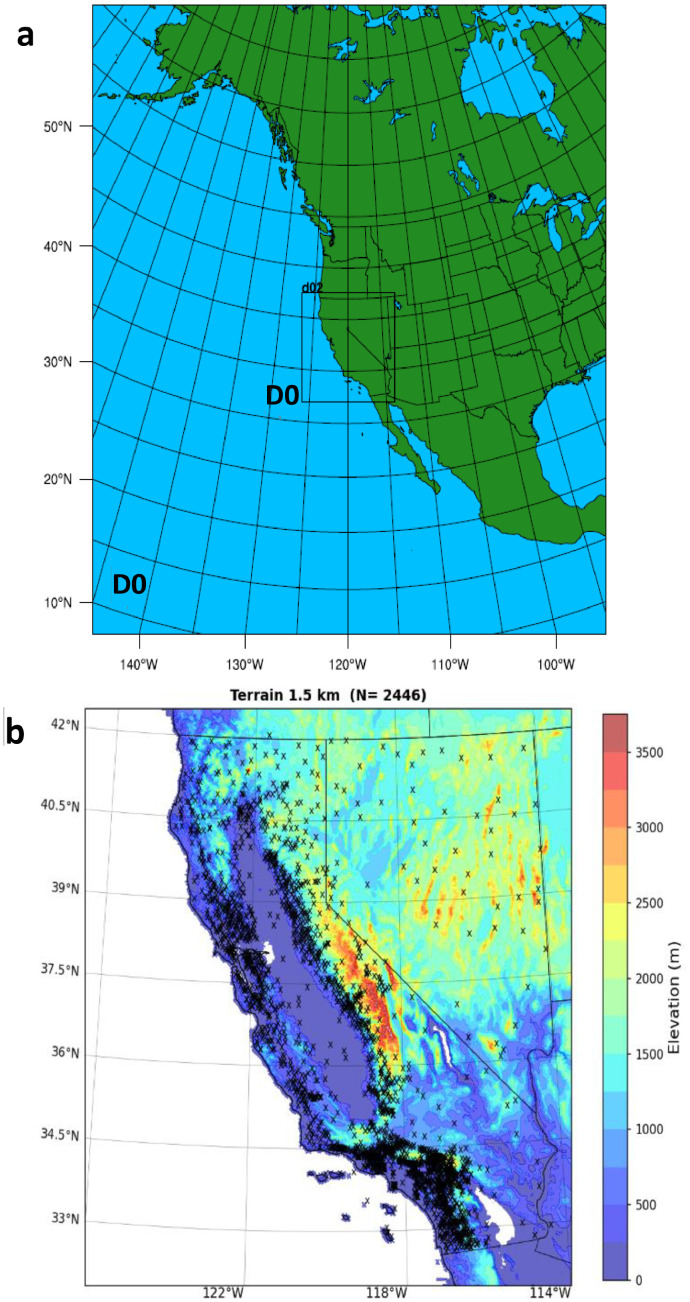
a) WRF configuration includes two-way nested grids D01 and D02 with 7.5 km and 1.5 km grid spacing, respectively. b) Inner domain D02. Colors indicate terrain elevation (m) and “x” symbols show surface weather stations used in WRF validation (N=2446).(For interpretation of the references to color in this figure legend, the reader is referred to the web version of this article.).

WRF was configured with two nested grids with two-way interaction. The outer and inner grids had 7.5 km and 1.5 km grid spacing (Fig. 2a). Fig. 2b shows the 1.5 km horizontal domain of the study area and the distribution of 2446 weather stations that had full or partial observations during 1 December-28 February 1990–2021.

## 4 Experimental Design, Materials and Methods

*WRF multi-physics analysis.* We first conducted multi-physics simulations in order to find a suite of physical parameterizations and horizontal grid spacing that resulted in relatively good agreement with observations from weather stations over California and Nevada. We focus on simulations of surface wind speed (WS), air temperature (TA) and relative humidity (RH). The period of simulations to identify a good physics suite extended from 6 November 00 UTC to 10 November 23 UTC, 2018, which included the Camp Fire in northern California, the deadliest wildfire in California history [6]. While it would be ideal to develop a multi-physics analysis over a longer period and test various combinations of physical parameterizations, the computational costs were prohibitively high. It is also important to note that the creation of this downscaling dataset [11] was made possible in a research project with limited allocation of High-Performance Computing core hours and duration. We note, however, that this period included high fire-weather conditions not only over the Camp Fire area but also in southern California, where Santa Ana wind conditions were present. To compensate for the short period of analysis, validation was performed using observations from the entire 1.5 km grid domain, ensuring high spatial sampling.

A total of 1753 surface weather stations were assembled with nearly complete records of WS, TA and RH during 6-10 November 2018. Note that some weather stations may record these variables in non-standard heights. We used WRF WS (10 m), TA (2 m) and RH (2 m) and did not attempt to convert non-standard observations to the same heights in WRF.

WRF was configured with two nested grids with two-way interaction. The outer grid had a 7.5 km grid spacing and we tested the sensitivity of the results with inner grids with 2.0 km, 1.5 km and 1.0 km grid spacing. Fig. 2a shows the horizontal domains of the 7.5 km and 1.5 km grids. The inner grids with 2.0 km and 1.0 km covered similar domains as the 1.5 km grid. Fig. 2b shows the 1.5 km horizontal domain and the distribution of 2446 weather stations that had full or partial observations during 1 December-28 February 1990–2021.

The reason for choosing a 7.5 km grid covering a large domain (i.e., Pacific Ocean and North America) is as follows. The size of the 1.5 km domain is 745 × 765 grid points in the east-west, north-south directions, respectively. In order to find a configuration that maximizes the number of computer cores available for multi-tasking and load balancing*,* the 7.5 km grid needs to be comparable in size to the 1.5 km grid. Thus, the size of the 7.5 km grid is 803 × 880 in the east-west, north-south directions, respectively. We tested 29 different combinations of physics parameterizations for surface layer physics, land-surface model, planetary boundary layer processes and grid resolutions such that:

• 26 combinations with 2.0 km horizontal grid spacing and 55 vertical levels.
• 1 combination with 2.0 km horizontal grid spacing and 32 vertical levels.
• 1 combination with 1.5 km horizontal grid spacing and 55 vertical levels.
• 1 combination with 1.0 km horizontal grid spacing and 55 vertical levels.

The other physical parameterizations were fixed: microphysics (Hong et al. 2006), radiation transfer (Iacono et al. 2008), cumulus parameterization in the 7.5 km grid and turned off in the 1.5 km grid (Zhang et al. 2011). Table 2, Table 3 show specific details of the combinations.

**Table 2:** WRF multi-physics configurations. Combinations were done with different parameterizations of surface layer physics (X), land surface model (Y) and planetary boundary layer physics (Z). Number in parenthesis represent flags used in the WRF namelist file. Multi-physics combinations are show in Table 2 with X-Y-Z codes. Additional details about the physics and references are available at the NCAR WRF website (https://www2.mmm.ucar.edu/wrf/users/physics/phys_references.html).

Surface layer physics X	Land surface model	Planetary boundary layer physics
X	Y	Z
Revised MM5 (1)	Unified Noah (2)	Yonsei University (1)
Eta similarity (2)	Noah-MP land surface (4)	Mellor-Yamada-Janjic (2)
QNSE (4)	Pleim-Xiu (7)	Quasi-normal scale elimination (4)
MYNN (5)		Mellor-Yamada-Nakanishi level2.5 (5)
		Asymmetric convection mode 2 (7)
		Bougeault-Lacarrere (8)
		Shin-Hong scale aware

**Table 3:** Statistics between WRF multi-physics simulations and 1753 weather station observations in California and Nevada. Statistics were computed between hourly time series from 6 November 00UTC to 10 November 23 UTC, 2018. Code 2-2-2* had 32 vertical levels, all others had 55 vertical levels. All configurations had 2-km horizontal grid spacing, except for 2-4-5-1.5 and 2-4-5-1.0, which had 1.5 km and 1.0 km horizontal grid spacing, respectively. Bold indicates the 2-4-5-1.5 configuration selected in the WRF downscaling.

Code	Wind speed			Air temperature			Relative humidity		
	Bias	RMS error	Corr.	Bias	RMS error	Corr.	Bias	RMS error	Corr.
1-2-1	−1.85	3.13	0.55	−0.72	3.2	0.9	5.61	16.29	0.84
1-2-11	−1.86	3.14	0.55	−0.72	3.2	0.9	5.83	16.38	0.84
1-2-5	−1.75	3.01	0.54	−0.78	3.22	0.9	6.3	16.3	0.85
1-2-7	−1.89	3.1	0.55	−0.91	3.28	0.9	7.18	16.8	0.85
1-2-8	−1.82	3.07	0.54	−1.28	3.46	0.9	6.48	16.93	0.84
1-4-1	−1.34	2.48	0.58	−0.66	2.86	0.92	5.75	16.13	0.85
1-4-11	−1.35	2.49	0.58	−0.65	2.87	0.92	5.98	16.23	0.85
1-4-5	−1.24	2.38	0.58	−0.69	2.88	0.92	6.26	16.07	0.85
1-4-7	−1.37	2.46	0.58	−0.84	2.92	0.92	7.15	16.63	0.85
1-4-8	−1.33	2.49	0.57	−1.25	3.11	0.92	6.63	16.65	0.85
1-7-1	−1.27	2.44	0.57	−0.82	3.01	0.91	−3.43	14.65	0.87
1-7-11	−1.28	2.44	0.57	−0.81	3.01	0.91	−3.13	14.54	0.87
1-7-5	−1.17	2.33	0.56	−0.84	3	0.92	−2.82	14.42	0.87
1-7-7	−1.29	2.42	0.57	−0.95	3.05	0.91	−1.15	14.03	0.87
1-7-8	−1.27	2.46	0.56	−1.19	3.17	0.91	−2.84	15	0.86
2-2-2	−1.7	2.98	0.57	−1.43	3.3	0.91	5.58	15.89	0.85
2-2-2*	−1.71	2.99	0.57	−1.44	3.3	0.91	5.58	15.88	0.85
2-2-5	−1.62	2.9	0.56	−1.58	3.37	0.91	6.81	16.14	0.85
2-2-8	−1.73	2.99	0.56	−2.13	3.72	0.9	7.21	16.84	0.84
2-4-5	−1.09	2.29	0.59	−0.75	2.91	0.92	6.07	15.91	0.85
2-4-8	−1.23	2.43	0.59	−1.32	3.17	0.91	6.5	16.54	0.85
2-7-5	−1.04	2.24	0.58	−1.13	2.9	0.93	−2.44	13.74	0.88
4-2-4	−1.73	2.96	0.57	−0.77	2.91	0.92	3.55	15.06	0.85
4-4-4	−1.19	2.33	0.59	−0.62	2.85	0.92	4.92	15.52	0.85
5-2-5	−1.74	2.97	0.55	−0.51	3.3	0.9	5.52	15.85	0.85
5-4-5	−1.22	2.35	0.58	−0.67	2.89	0.92	6.32	16.14	0.85
5-7-5	−1.13	2.3	0.57	−0.97	3	0.92	−2.44	14.24	0.87
**2-4-5-1.5**	−**0.96**	**2.11**	**0.61**	−**0.64**	**2.85**	**0.92**	**5.66**	**15.51**	**0.86**
2-4-5-1.0	−0.95	2.12	0.62	−0.62	2.83	0.92	5.54	15.45	0.86

**Fig. 3:**
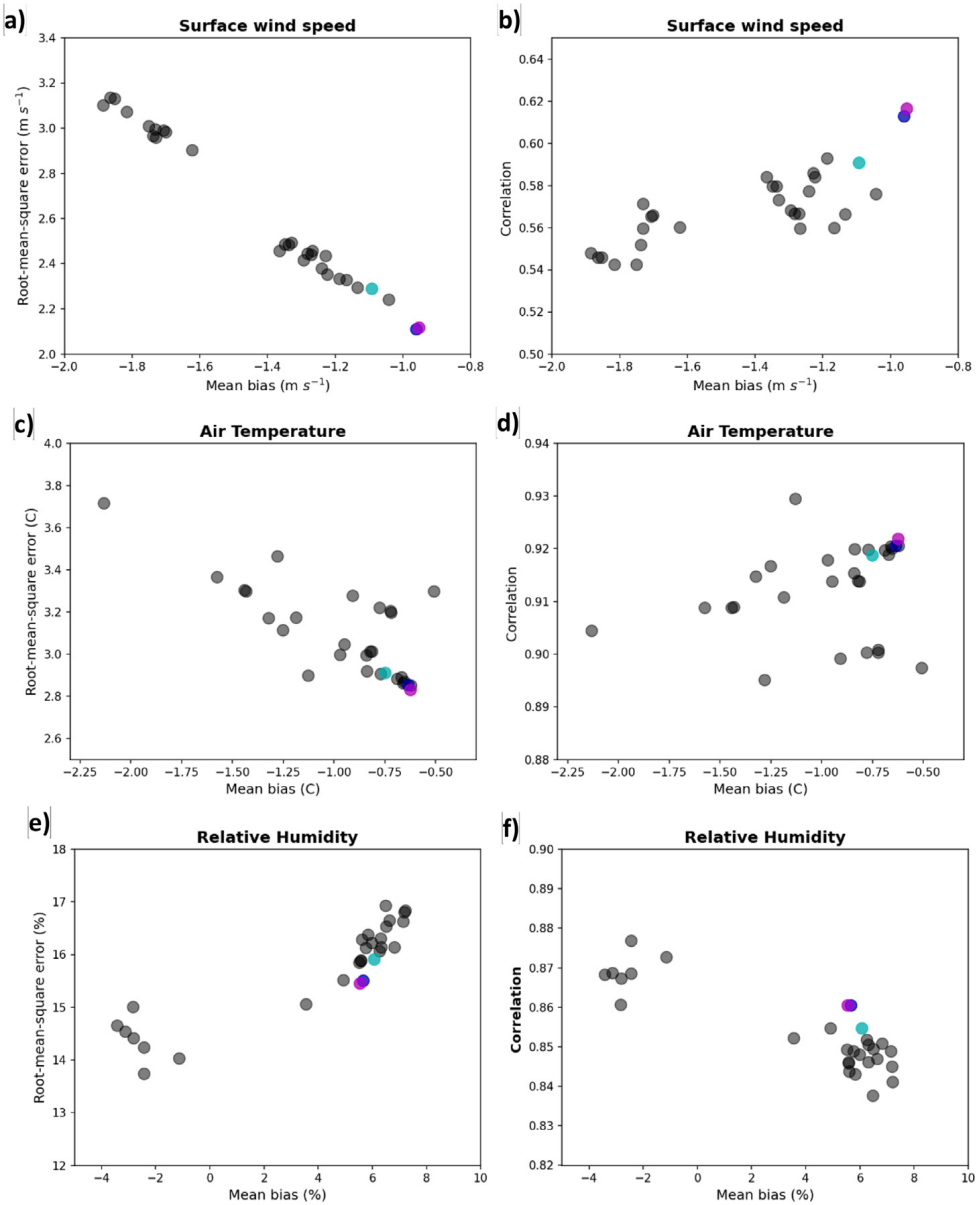
Statistics between multi-physics WRF simulations and 1753 weather station observations in California and Nevada. Statistics were computed between hourly time series from 6 November 00UTC to 10 November 23 UTC, 2018. Gray circles show statistics for different configurations of WRF physics with 2 km horizontal grid spacing. Cyan circles show statistics for WRF 245 configuration with 2-km grid spacing; blue and magenta circles show statistics for WRF 245 configuration with 1.5-km and 1.0-km grid spacing, respectively. Mean bias is defined as observation minus model data. Panels show statistics for a-b) wind speed, c-d) air temperature and e-f) relative humidity. See text for additional information about WRF multi-physics.(For interpretation of the references to color in this figure legend, the reader is referred to the web version of this article.).

For each combination of parameterizations, we aggregated all hourly time series of weather station observations and corresponding WRF nearest gridpoint time series and computed mean bias, root-mean square error and correlation. Fig. 3 shows scatter plots of the statistics. Interestingly, there is a nearly linear correspondence between mean bias and root-mean square error (and correlation) for WS (Fig. 2 a, and b). Certain groups of combinations significantly underperform relative to others; see also Table 2. Importantly, we note that the combination (named 2-4-5) of Eta similarity surface layer physics, Noah-MP land-surface model and Mellor-Yamada-Nakanishi planetary boundary layer physics with 2.0 km grid (cyan circle) is among the best performances. Further increasing the resolution to 1.5 km (blue circle) and 1.0 km (magenta circle) grids result in additional increases in performance. While there is very little difference between 1.5 km and 1.0 km grids, the 1.5 km shows additional gains in performance relative to the 2.0 km. This is expected, since high model resolution is able to better represent surface winds in regions of complex topography^17^. Somewhat similar relationships are found for TA (Fig. 2 c and d), although not as linear. The statistics for RH (Fig. 2 e, f) show two distinct groups of combinations and the 2-4-5 combination is among the best performances. In summary, the WRF 2-4-5 combination with 1.5 km inner grid exhibited about 10–20 % smaller biases and root-mean square errors in WS and TA than the 2-4-5 combination with 2.0 km grid and, therefore, was selected for the 30-yr high-resolution downscaling meteorological dataset [11] (named WRF-CALNEV).

The multi-physics experiments and 30-yr downscaling simulations were performed using the *High-Performance Computing* resources from the Lawrence Berkeley National Laboratory (LLNL, https://hpc.llnl.gov) and NASA *High-End Computing Capability* (HECC, https://www.nas.nasa.gov/hecc).

**WRF 30-yr high-resolution downscaling.** Based on the results from the multi-physics analysis, the 30-yr high-resolution downscaling was performed with the following methodology. The ERA5 reanalysis [12] was used to derive initial and boundary conditions for WRF simulations. ERA5 reanalysis was downloaded from the National Center for Atmospheric Research (NCAR, https://rda.ucar.edu/datasets/d633000/) WRF was run in seasonal batches: 30 November 00 UTC – 1 March 00 UTC, 28 Feb 00 UTC – 1 June 00 UTC, 31 May 00 UTC – 1 September 00 UTC and 31 October 00 UTC – 1 December 00 UTC, such that the 30-yr records extends from 1 January 00 UTC 1991 to 28 February 00 UTC 2021. The first day of each seasonal batch was discarded. Output frequency for the 1.5 km domain was 1-h. The choice of running WRF in seasonal batches, as opposed to yearly batches or continuous multi-year simulations, was based on the computational resources available at the time as well as efficiency in finishing up simulations in a reasonable time during periods of heavy usage of the High-Performance Computing systems. Additionally, the research project under which this dataset [11] was created had a total duration of three years.

Note that the short spinup time of 1-day may affect trend analysis of some variables such as soil moisture and precipitation, particularly during the rainy season. However, we recall that the primary goal of providing this dataset [11] to the research community is for weather and climatological studies that do not rely on long spinup times. Furthermore, grid nudging in the 7.5 km grid ensures that the simulations follow the ERA5 reanalysis throughout the seasonal simulations and relies on the physical and dynamical representations in WRF to downscale to 1.5 km. Additional details about model spinup are discussed in the technical validation.

Lateral and lower boundary conditions were updated every 3-h throughout the simulations. Grid nudging towards the reanalysis was applied in winds, temperature and specific humidity in the 7.5 km grid only, including within the planetary boundary layer. Another advantage of having the 7.5 km grid covering a large domain (Fig. 1a), in addition to computational efficiency, is that the grid nudging ensures that large-scale waves in the coarse domain follow the ERA5 reanalysis throughout the seasonal simulations and the information is passed through the lateral boundaries of the 1.5 km grid.

The configuration used numerical parameterizations for microphysics (Single moment 6-class) [13], long-wave and short-wave radiation transfer (RRTMG) [14], Noah land surface model with multi-parameterization (Noah-MP) [15], surface layer physics and planetary boundary layer (MYNN) [16]. Following the practice of most studies^21,22^, cumulus parameterization (Tiedtke scheme) [17] was applied only on the 7.5 km grid. This combination of WRF parameterizations was also based on our previous research, [18,2] where we have performed extensive comparisons between WRF simulations and surface weather observations. Table 4 shows additional details used in the WRF configuration.

**Table 4:** Details of WRF-CALNEV grid and physics configurations.

WRF Grid Configuration	Lambert	
Map Projection		
Reference Latitude, Longitude	40°N, 119.8°W	
True Latitudes	30°N, 60°N	
Standard Longitude	120°W	
Grid ratio	1:5	
Static files	Default	
Domains	D01	D02
Horizontal grid spacing	7.5 km	1.5 km
Size (grid points)	831 E-W; 881 N-S	746 E-W; 766 N-S
Vertical levels	55	55
WRF Physics Configuration		
	D01 (7.5 km)	D02 (1.5 km)
Boundary Conditions Update	3 h	3 h
Output history	24 h	1 h
Sea surface temperature update	3 h	3 h
Time step (fixed)	30 s	6 s
Microphysics	Single moment 6 class (Hong et al. 2006)	Single moment 6 class (Hong et al. 2006)
Radiation	RRTMG (Iacono et al. 2008)	RRTMG (Iacono et al. 2008)
Radiation update	10 min	10 min
Surface layer physics	Eta similarity (Janjic et al. 2002)	Eta similarity (Janjic et al. 2002)
Land surface model	Noah-MP (He et al. 2023)	Noah-MP (He et al. 2023)
Planetary boundary layer	MYNN (Olson et al. 2019)	MYNN (Olson et al. 2009)
Cumulus parameterization	Tiedtke scheme (Zhang et al. 2011)	No
Cumulus radiation feedback	Yes	Yes
Slope dependent radiation	No	Yes
Grid nudging, interval	Yes, 3 h	No
Nudging coefficient	0.0003	No
Nudging Variables	Winds, temperature, specific humidity	No
Grid nudging in PBL	Yes	No

Note that the short spinup time of 1-day may affect trend analysis of some variables such as soil moisture, particularly during the rainy season, and precipitation. However, we recall that the primary goal of providing this dataset [11] to the research community is for weather and climatological analyses that do not rely on long spinup times. Furthermore, grid nudging only in the 7.5 km grid ensures that the simulations follow the ERA5 reanalysis throughout the seasonal simulations and relies on the physical and dynamical representations in WRF to downscale to 1.5 km.

**Fig. 4:**
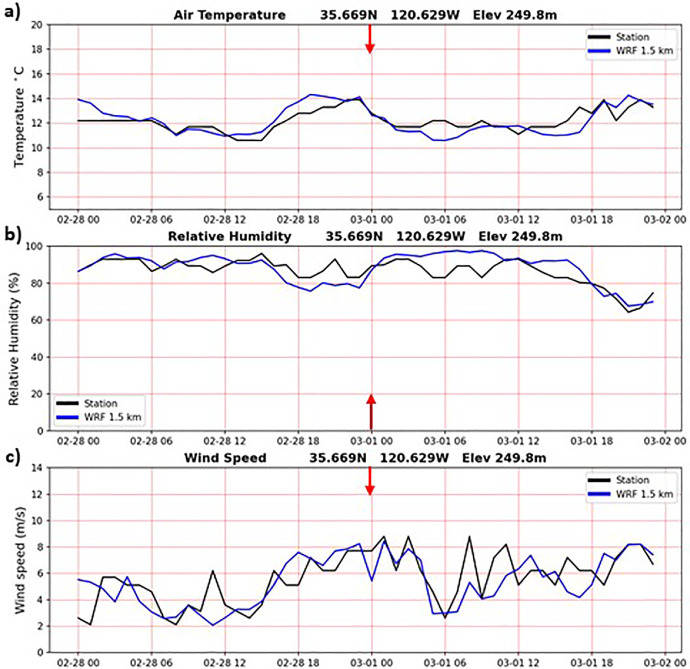
Times series from surface weather station (black) and WRF 1.5 km nearest grid point (blue). WRF times series connect two seasonal downscaling batches: 1) 11/30/1990 – 02/28/1991 and 2) 02/28/1991-05/31/1991. The 1-day spinup in the second batch (02/28/1991) was dropped off from the time series. Red arrows indicate the connecting days between the two seasonal downscaling batches. a) air temperature (C), b) relative humidity (%) and c) wind speed (m/s). Location: 35.669N, 120.629W, elevation 249.8 m.(For interpretation of the references to color in this figure legend, the reader is referred to the web version of this article.).

**Fig. 5:**
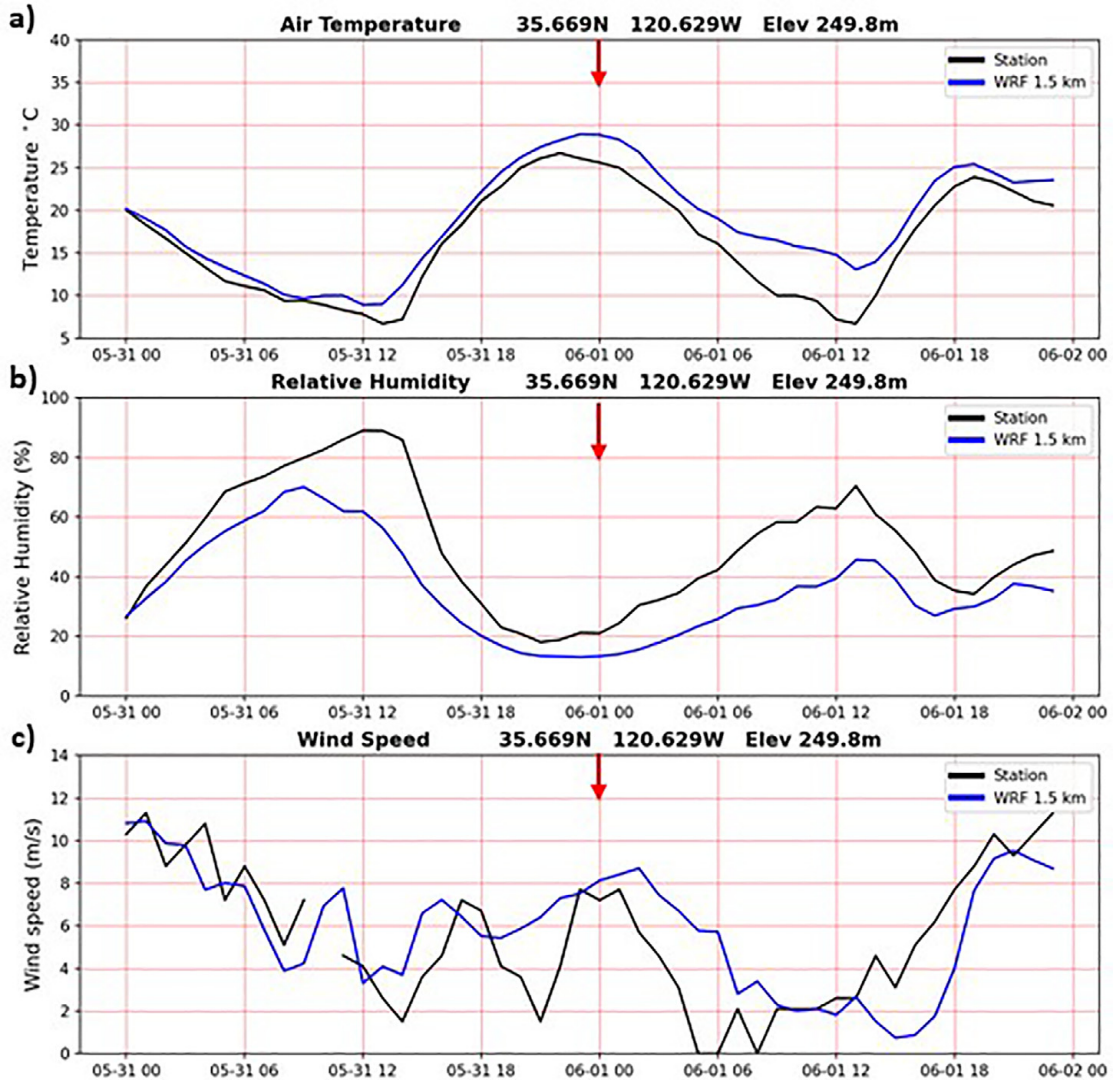
As in Fig. 3, but for seasonal batches: 1) 02/28/1991 – 05/31/1991 and 2) 05/31/1991 – 08/31/1991. The 1-day spinup in the second batch (05/31/1991) was dropped off from the time series.

**Fig. 6:**
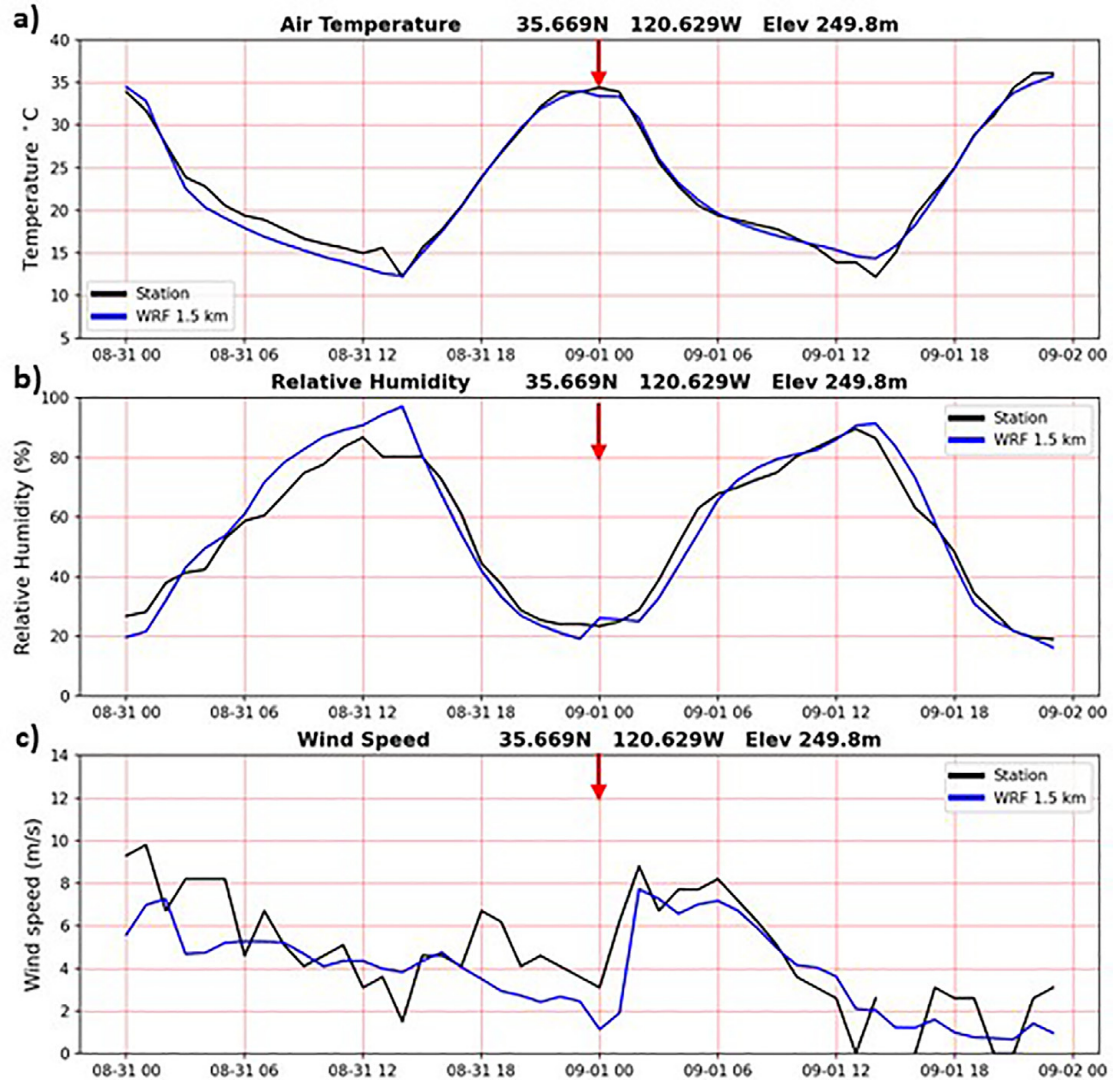
As in Fig. 3, but for seasonal batches: 1) 05/31/1991 – 08/31/1991 and 2) 08/31/1991-11/30/19911. The 1-day spinup in the second batch (08/31-1991) was dropped off from the time series.

**Fig. 7:**
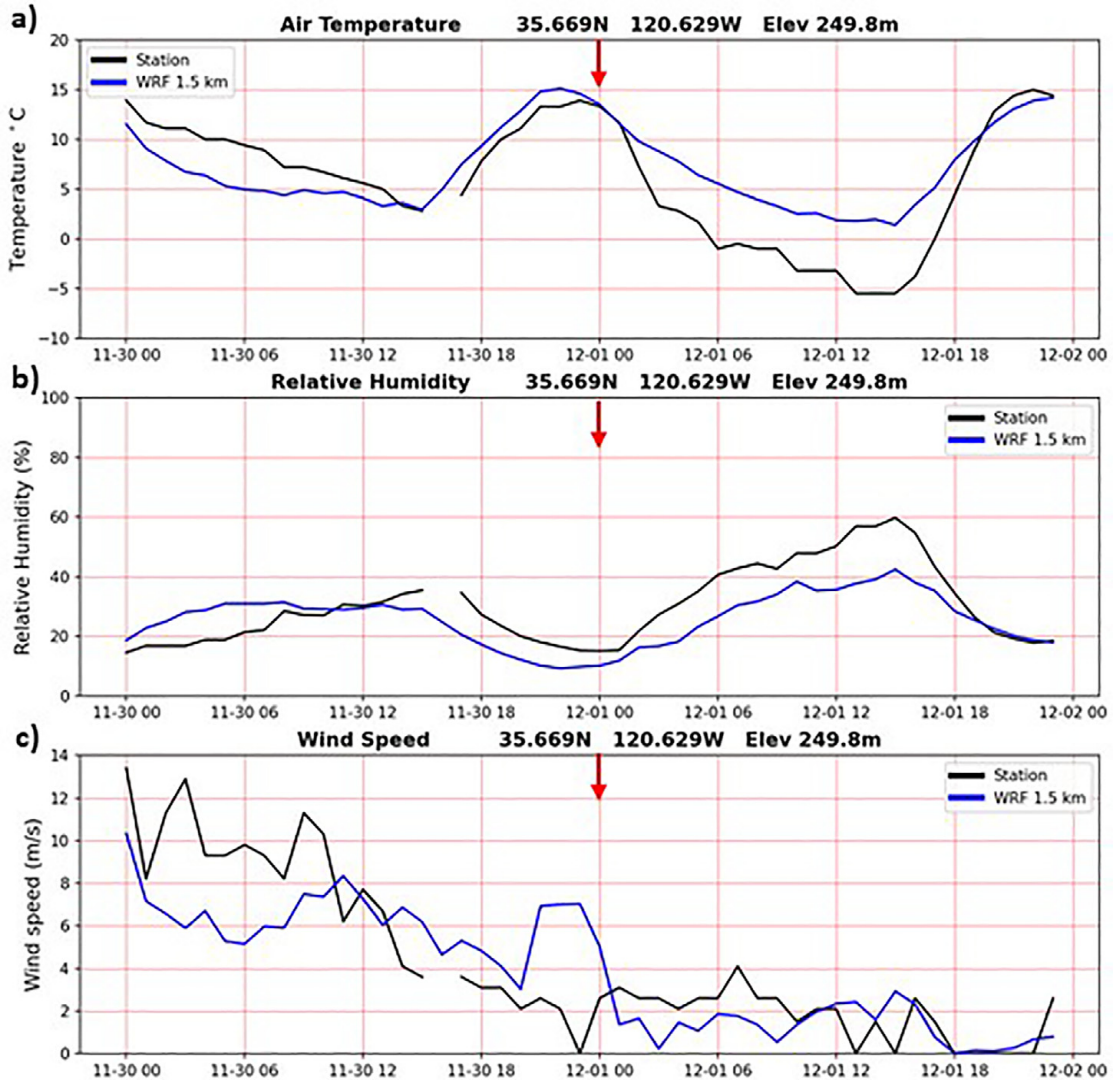
As in Fig. 3, but for seasonal batches: 1) 08/31/1991 – 11/30/1991 and 2) 11/30/1991 – 02/29/1992. The 1-day spinup in the second batch (11/30/1991) was dropped off from the time series.

In addition to the validation results during the Camp Fire period presented above, the WRF-CALNEV downscaling dataset [11] was validated in several different ways. The importance of 1-day spinup was examined as follows. Fig. 4 shows TA, RH and WS observations from a weather station located in Paso Robles, California, one of the few stations with nearly complete records in the 30-yr period of this study. WRF 1.5 km time series from the nearest grid point are also plotted, connecting the end of the 11/30/1990-02/28/1991 seasonal batch with the start of the 02/28/1991-05/31/1991 seasonal batch, after discarding the 1-day spinup of 02/28/1991. Aside from random errors, more evident in WS, there is no discontinuity associated with the 1-day spinup, as indicated by the red arrows. In cold start simulations, such as the one adopted here, it takes a few hours for surface atmospheric variables to reach a new equilibrium. Similarly, Fig. 5, Fig. 6, Fig. 7 show similar plots of time series connecting other seasonal batches, confirming that 1-day spinup has no significant impacts on surface TA, RH and WS.

**Fig. 8:**
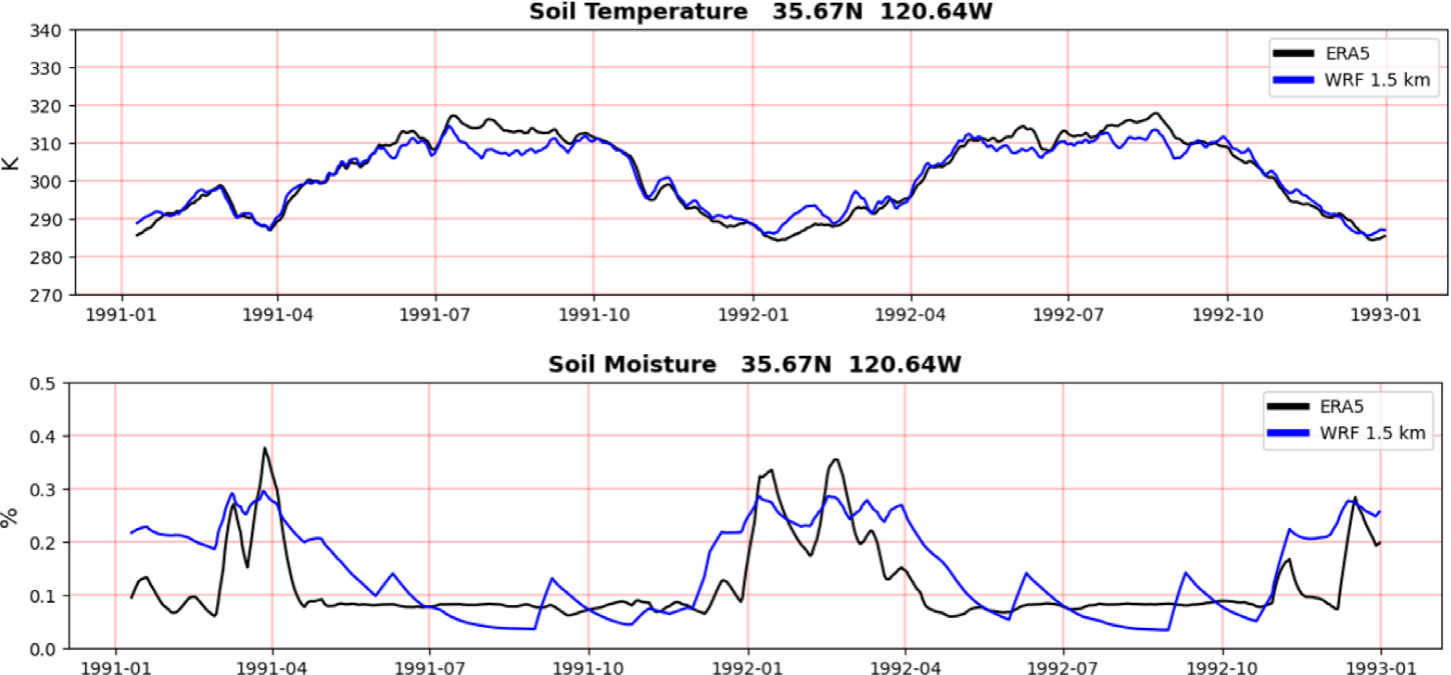
a) Soil temperature from ERA5 reanalysis (0–7 cm, black) and WRF 1.5-km (0–10 cm, blue). b) Soil moisture from ERA5 reanalysis (0-7 cm, black) and WRF 1.5-km (0–10 cm, blue). Location: 35.67N, 120.64W. Time series are sampled once a day (00UTC) and a 10-day moving average was applied to the time series to filter high frequency variations. Asterisks show initialization times.(For interpretation of the references to color in this figure legend, the reader is referred to the web version of this article.).

Fig. 8 shows the importance of 1-day spinup on land surface variables. Since the WRF model is initialized with ERA5 data, the purpose here is to assess how the WRF 1.5 km land surface variables (0–10 cm, blue) deviate from the ERA5 surface layer (0–7 cm, black) background over time. We recall that grid nudging is applied only in the WRF 7.5 km grid. The time series from the WRF 1.5 km seasonal batches have been concatenated from 01/01/1991 to 01/01/1993. Both time series are sampled once a day (00 UTC) and a 10-day moving average was applied to emphasize long-term changes. Despite the difference in spatial resolutions, the WRF 1.5 km soil temperature (Fig. 8a) tracks the ERA5 soil temperature remarkably well and the 1-day spinup does not seem to have any appreciable influence. In contrast, as expected, soil moisture (Fig. 8b) shows large increases at the start of the seasonal batches (indicated by *), suggesting that a longer spinup than 1-day is required for hydrometeorological processes.

**Fig. 9:**
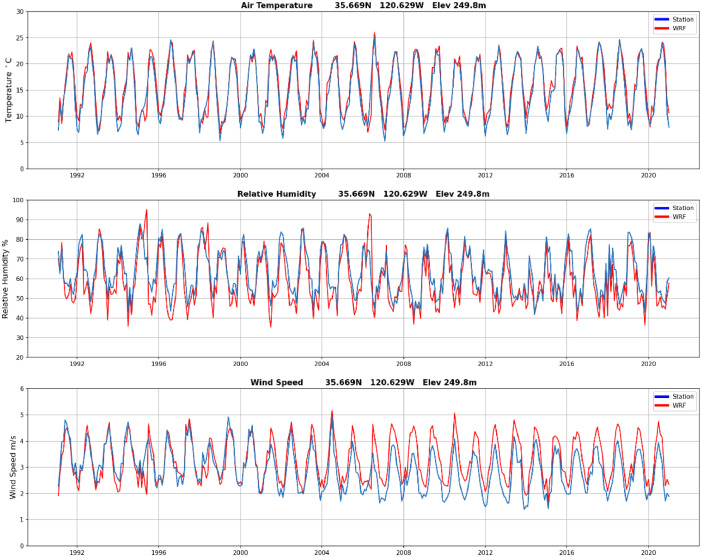
Comparison of observed (blue) and WRF 1.5 km (red) monthly time series of a) air temperature (C), b) relative humidity (%) and wind speed (m s^-1^) during 1991–2020. Station location: Paso Robles Municipal Airport (35.669 N, 120.629W and elevation 249. 8 m).(For interpretation of the references to color in this figure legend, the reader is referred to the web version of this article.).

To obtain a long-term perspective on the performance of the dataset [11], Fig. 9 shows monthly time series of TA, RH and WS (blue lines) from the long-term monitoring weather station in Paso Robles, California. WRF 1.5 km time series from the nearest grid point are also shown (red lines). TA in this location (Fig. 9a) shows a regular annual cycle between 7 and 26 C. TA from the WRF downscaling shows excellent agreement with observed values. Likewise, observed RH values range between 40–89 % (Fig. 9b), while WRF 1.5 km ranges between 39 and 95 %. WRF 1.5 km is highly correlated with observed values. Observed WS also shows a regular seasonal cycle, which is realistically represented in the WRF 1.5 km, although it is clear that maximum and minimum monthly wind speeds in this location are over and under predicted by the model, respectively. It is also interesting to note that the WRF 1.5 km is able to represent short-term variations on 1-2 months, especially in TA and RH. This agreement between observations and model was also found in other locations, although, due to short observational records or terrain influences, it is somewhat less clear.

**Fig. 10:**
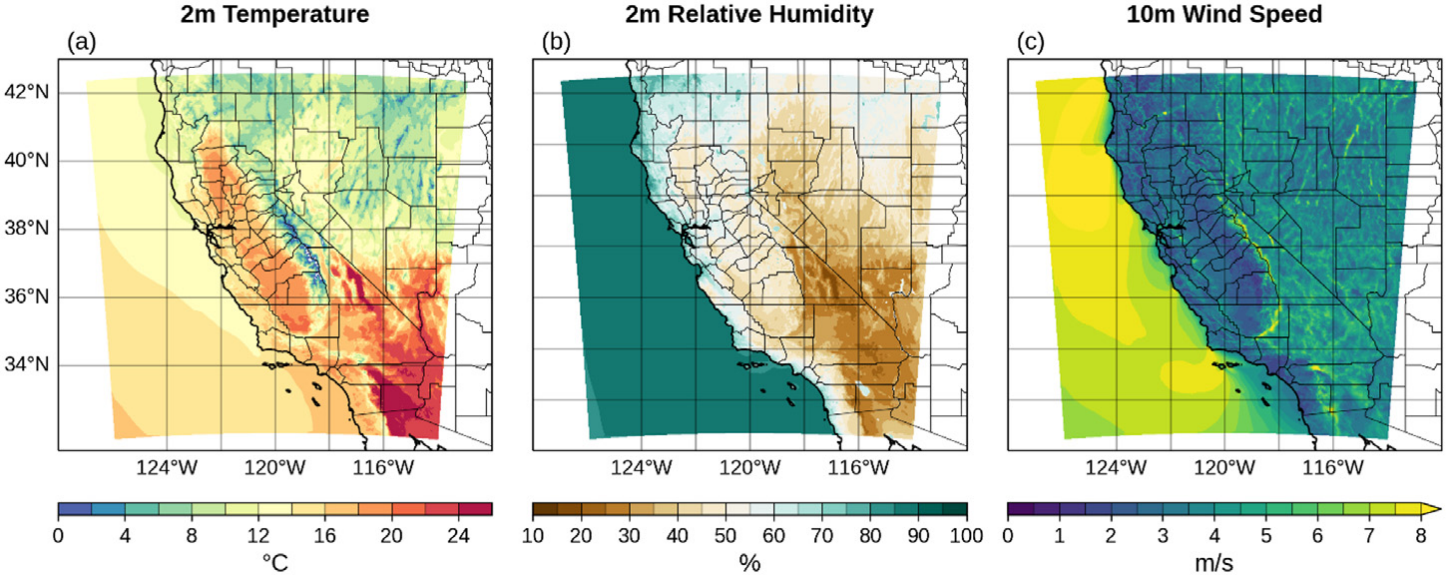
Climatology of a) air temperature at 2 m (C), b) relative humidity at 2 m (%) and c) wind speed at 10 m (m s^-1^). Period: 1 January-31 December, 1991–2020.

**Fig. 11:**
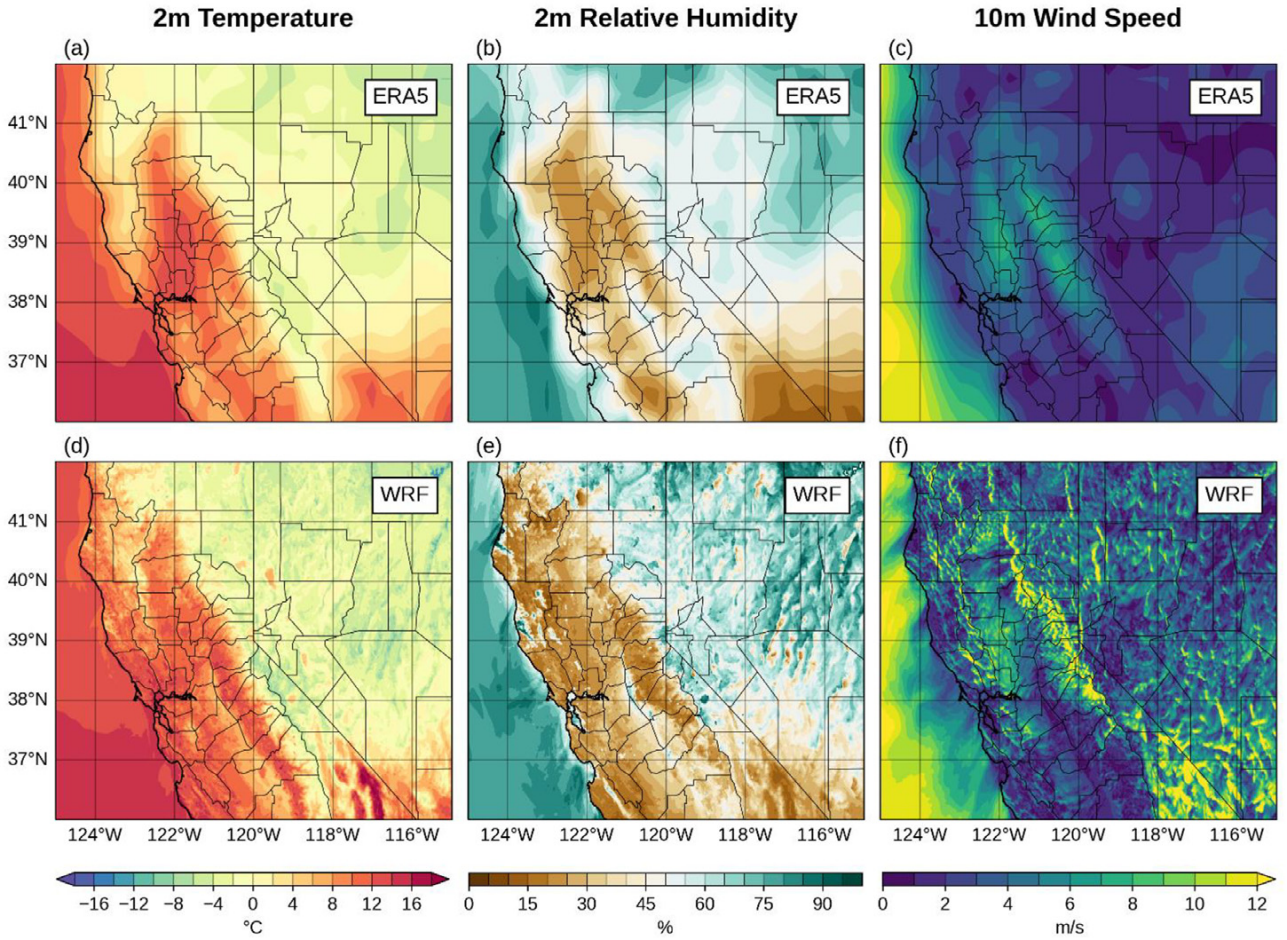
Top row shows a) air temperature at 2 m (C), b) relative humidity at 2 m (%) and c) wind speed at 10 m (m s^-1^) from ERA5 reanalysis during 8 November 2018 at 15:00 UTC. Bottom row shows d) air temperature at 2 m (C), e) relative humidity at 2 m (%) and f) wind speed at 10 m (m s^-1^) from WRF 1.5 km downscaling during 8 November 2018 at 15:00 UTC.(For interpretation of the references to color in this figure legend, the reader is referred to the web version of this article.).

The long-term means of TA, RH and WS (Fig. 10) show consistent and expected spatial variations across California and Nevada with warm and dry desert regions, warm central valley, cooler and windy high mountain areas. The added value of the downscaling dataset is illustrated in Fig. 11, which compares TA, RH and WS from ERA5 reanalysis and WRF 1.5 km during Camp Fire on 8 November 2018 (15:00 UTC). Since WRF was driven by ERA5 reanalysis, it is anticipated that all fields are internally consistent. Importantly, WRF 1.5 km shows much richer spatial structures than ERA5, especially in surface winds, which are crucial to monitor extreme fire weather conditions.

Additional validation analyses used hourly observations of TA, RH and WS from 2446 weather stations over California and Nevada during 1 January-31 December 1990–2020. The weather stations are available from Mesowest data providers (synopticdata.com, https://www.westernweathergroup.com, https://mesowest.utah.edu). Quality control consisted in 1) eliminating surface weather stations with less than six months of data (4320 data points) and 2) eliminating suspicious data values outside 1.5 * IQ, where IQ was the interquartile range. The selected network of surface weather stations included:

1) Global long-term monitoring weather stations (38 stations) assembled by the Met Office Hadley Centre (https://www.metoffice.gov.uk/hadobs/hadisd/) and available through the caladapt server (https://cal-adapt.org/). These stations had nearly complete record of hourly observations during the 30-year period of the downscaling.
2) Surface stations from the Pacific Gas & Electric (PG&E; 831 stations), Southern California Edison (SCE, 878), San Diego Gas & Electric (SDGE, 213 stations) and Remote Automatic Weather Stations (RAWS, 486 stations) network. This choice of data was specially motivated by sampling weather observations in high elevation mountain areas, in order to further estimate the accuracy of WRF simulations.

**Fig. 12:**
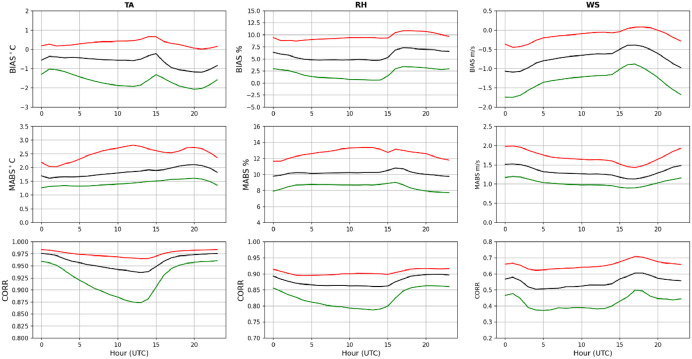
Validation: columns show statistics between hourly time series of observed and WRF 1.5 km simulations of TA (left), RH (middle) and WS (right). Rows show mean bias (top), mean absolute error (middle) and correlation (bottom). Statistics are computed at each surface weather station separately and then aggregated. Black lines show median and interquartile ranges with red and green lines. Horizontal axes show hours in UTC.(For interpretation of the references to color in this figure legend, the reader is referred to the web version of this article.).

A quantitative assessment of the quality of the WRF 1.5 km dataset [11] was derived by computing mean biases, mean absolute errors and correlations between hourly time series of TA, RH and WS from weather stations and model. The statistics were computed at each location separately and then aggregated by computing means and interquartile ranges of the statistics for easy presentation of the results (Fig. 12). Statistics for TA (Fig. 12 left column) show relatively small diurnal variations in mean biases but indicate a warm model bias ranging between −0.5 C and −1.0 C. The interquartile ranges of mean bias vary between −2.0 C and 0.8 C. Mean absolute errors also show small diurnal variations with mean between 1.6 and 2.0 C and interquartile ranges between 1.3–2.8 C. Mean correlations range between 0.94 and 0.975 (interquartile range 0.875-0.980). Mean biases in RH (Fig. 12 middle column) show similar weak diurnal variations varying between 5.0 and 7.5 % (interquartile ranges: 2.5–11 %). Additionally, mean absolute errors are approximately 10 % throughout the day with interquartile ranges between 8.0 and 13 %. Mean correlations are about 0.85–0.90. Unlike TA and RH, mean biases for WS (Fig. 12 right column) show that WRF 1.5 km tends to over predict winds. Mean biases are more negative in late afternoon (∼2:00 UTC) and least negative in early morning (∼17:00 UTC) (mean biases: −1.0 m s^-1^ and −0.5 m s^-1^; interquartile ranges: -1.7 m s^-1^ and 0.0 m s^-1^). Interestingly, mean absolute errors do not show strong diurnal variations varying between 1.2 and 1.5 m s^-1^ (interquartile ranges: 1.0-2.0 m s^-1^). Somewhat expectedly, mean WS correlations between observations and WRF 1.5 km are smaller than for TA and RH varying between 0.5 and 0.6 (interquartile ranges: 0.4–0.7).

**Fig. 13:**
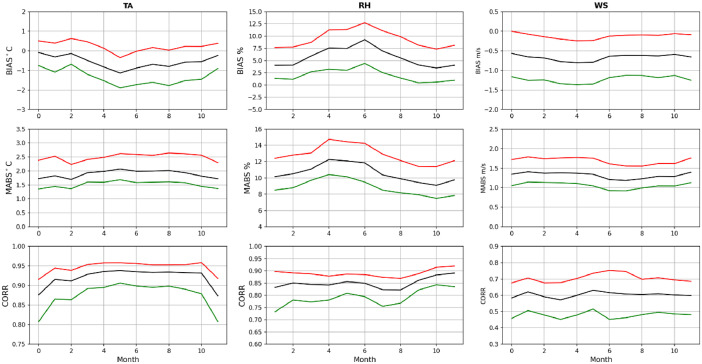
Validation: columns show statistics between monthly time series of observed and WRF 1.5 km simulations of TA (left), RH (middle) and WS (right). Rows show mean bias (top), mean absolute error (middle) and correlation (bottom). Statistics are computed at each surface weather station separately and then aggregated. Black lines show median and interquartile ranges with red and green lines. Horizontal axes show months of the year.(For interpretation of the references to color in this figure legend, the reader is referred to the web version of this article.).

Validation of the downscaling dataset was further evaluated by computing mean biases, mean absolute errors and correlations between time series of TA, RH and WS from weather stations and model aggregated by months of the year. As before, means and interquartile ranges of the individual statistics were calculated for easy interpretation. The mean bias in TA is slightly higher in late spring than in other months (Fig. 13 left column) ranging between 0.0 C and −1.0 C (interquartile ranges: −2.0 C and 0.8 C). Mean absolute errors in TA do not vary significantly throughout the year and are approximately 2.0 C (interquartile ranges: 1.5–2.5 C). TA correlations, however, are lowest in winter months (∼0.87) and nearly constant in other seasons (0.94) (interquartile ranges: 0.80–0.95). Interestingly (Fig. 13 middle column), the downscaling dataset has highest RH mean bias in summer (9.0 %) and lowest in winter months (2.8 %) (interquartile ranges: 0.0–12.5 %). Mean absolute errors in RH are highest (12 %) in the spring and lowest in late fall (8 %) (interquartile ranges: 8.0–14 %). Mean RH correlations do not vary appreciably throughout the year varying between 0.85 and 0.90 (interquartile ranges: 0.75–0.92). Statistics for WS (Fig. 13 right column) do not exhibit large seasonal variations. Mean biases vary between −0.8 m s^-1^ and −0.4 m s^-1^ (interquartile ranges: -1.4 m s^-1^ and 0.0 m s^-1^). Mean absolute errors are about 1.3 m s^-1^ on average (interquartile ranges: 1.0–1.8 m s^-1^). Mean correlations are approximately 0.6 (interquartile ranges: 0.35–0.75).

## Limitations

By design, this downscaling dataset was created focusing on weather rather than climate simulations, when sufficient model spinup is required. The dataset [11] covers the states of California and Nevada in the United States and is a unique resource for a number of potential applications such as analysis of fire-weather conditions, extreme events (e.g., heat waves, high winds etc.) and regional atmospheric circulation studies requiring high spatiotemporal resolutions.

The technical validation indicates that the WRF CALNEV dataset [11] can be a valuable resource for studies requiring high spatiotemporal resolutions focusing on weather scales, especially in resolving atmospheric circulations in complex terrain. Given the project and computational constraints under which this dataset was created, users should be mindful of its limitations. Users interested in climate simulations and hydrometeorological studies that necessitate prolonged model spinup should use the data with caution or consider alternate datasets.

## Ethics Statement

The authors have read and follow the ethical requirements for publication in Data in Brief and confirming that the current work does not involve human subjects, animal experiments, or any data collected from social media platforms.

## CRediT authorship contribution statement

C. Jones designed the WRF configuration and downscaling strategy and validation analysis. Downscaling computer jobs were performed by C. Jones and A. Bagley. D. Lucas helped with the Lawrence Livermore National Laboratory High-Performance Computing system, analysis and interpretation of the results. C. Thompson helped downloading and preparing the surface weather station observations used in the validation analysis.

## Data Availability

World Data Center for ClimateA 30-yr high-resolution weather research and forecasting model downscaling data over California and Nevada (Original data).
